# Supplementary material for: Mechanisms governing the pioneering and redistribution capabilities of the non-classical pioneer PU.1
Source: Nat Commun. 2020 Jan 21;11:402. doi: 10.1038/s41467-019-13960-2 (PMC6972792; doi:10.1038/s41467-019-13960-2)
Supplement: Supplementary file 7 — Source data [file 41467_2019_13960_MOESM7_ESM.zip › Source_Data/FigureS2/FigureS2B_MotifScanOutput/KmeansCluster6/homerResults/motif33.similar.html]

motif33

## Information for motif33

C
A
T
G
A
C
T
G
C
T
G
A
C
T
G
A
A
T
C
G
C
G
T
A
G
A
C
T
C
T
A
G
A
G
C
T
T
A
C
G
A
G
T
C
  
Reverse Opposite:  

C
T
A
G
A
T
G
C
C
T
G
A
A
G
T
C
C
T
G
A
G
C
A
T
T
A
G
C
A
G
C
T
A
G
C
T
G
T
A
C
G
T
A
C
  

|  |  |
| --- | --- |
| p-value: | 1e-35 |
| log p-value: | -8.276e+01 |
| Information Content per bp: | 1.756 |
| Number of Target Sequences with motif | 84.0 |
| Percentage of Target Sequences with motif | 2.55% |
| Number of Background Sequences with motif | 153.6 |
| Percentage of Background Sequences with motif | 0.36% |
| Average Position of motif in Targets | 95.3 +/- 43.0bp |
| Average Position of motif in Background | 97.0 +/- 78.8bp |
| Strand Bias (log2 ratio + to - strand density) | -0.5 |
| Multiplicity (# of sites on avg that occur together) | 1.01 |
| Motif File: | file (matrix) reverse opposite |

### Similar de novo motifs found

|  |  |  |  |  |  |  |  |
| --- | --- | --- | --- | --- | --- | --- | --- |
| Rank | Match Score | Redundant Motif | P-value | log P-value | % of Targets | % of Background | Motif file |
| 1 | 0.882 | A C T G G T A C C G T A A G T C C T G A C G A T T A G C A G C T A G C T G T C A A T G C G T C A C G A T | 1e-33 | -77.567798 | 2.34% | 0.32% | motif file (matrix) |
| 2 | 0.837 | C T G A C A T G G A T C C G T A G A T C C T A G C G A T A G T C G A C T A G C T T G C A A T G C | 1e-32 | -74.883876 | 1.76% | 0.16% | motif file (matrix) |
| 3 | 0.829 | A C G T T C G A T G C A A T C G C T G A A G T C T C A G A G C T A C T G T A G C | 1e-31 | -72.673156 | 2.40% | 0.37% | motif file (matrix) |
| 4 | 0.790 | C T A G G A T C C G T A A G T C C T G A C G A T A G T C A G C T C G A T G T C A A G T C T C G A A C G T A C T G | 1e-23 | -53.765401 | 1.49% | 0.18% | motif file (matrix) |
